# Liquid crystal-templated chiral nanomaterials: from chiral plasmonics to circularly polarized luminescence

**DOI:** 10.1038/s41377-022-00913-6

**Published:** 2022-07-14

**Authors:** Xuan Zhang, Yiyi Xu, Cristian Valenzuela, Xinfang Zhang, Ling Wang, Wei Feng, Quan Li

**Affiliations:** 1 School of Materials Science and Engineering, Tianjin University, 300350 Tianjin, China; 2 Institute of Advanced Materials and School of Chemistry and Chemical Engineering, Southeast University, 211189 Nanjing, China; 3 Advanced Materials and Liquid Crystal Institute and Chemical Physics Interdisciplinary Program, Kent State University, Kent, OH 44242 USA

**Keywords:** Nanoparticles, Liquid crystals

## Abstract

Chiral nanomaterials with intrinsic chirality or spatial asymmetry at the nanoscale are currently in the limelight of both fundamental research and diverse important technological applications due to their unprecedented physicochemical characteristics such as intense light-matter interactions, enhanced circular dichroism, and strong circularly polarized luminescence. Herein, we provide a comprehensive overview of the state-of-the-art advances in liquid crystal-templated chiral nanomaterials. The chiroptical properties of chiral nanomaterials are touched, and their fundamental design principles and bottom-up synthesis strategies are discussed. Different chiral functional nanomaterials based on liquid-crystalline soft templates, including chiral plasmonic nanomaterials and chiral luminescent nanomaterials, are systematically introduced, and their underlying mechanisms, properties, and potential applications are emphasized. This review concludes with a perspective on the emerging applications, challenges, and future opportunities of such fascinating chiral nanomaterials. This review can not only deepen our understanding of the fundamentals of soft-matter chirality, but also shine light on the development of advanced chiral functional nanomaterials toward their versatile applications in optics, biology, catalysis, electronics, and beyond.

## Abstract (web summary)

This review focuses on liquid crystal-templated chiral functional nanomaterials, including chiral plasmonic nanomaterials and chiral luminescent nanomaterials, introducing their underlying mechanisms, chiroptical properties, and potential applications.

## Graphical abstract

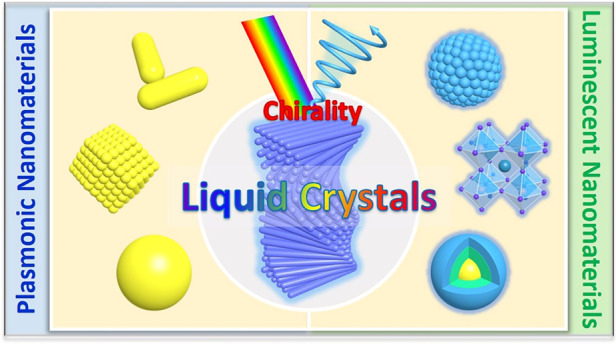

## Introduction

**Fig. 1:**
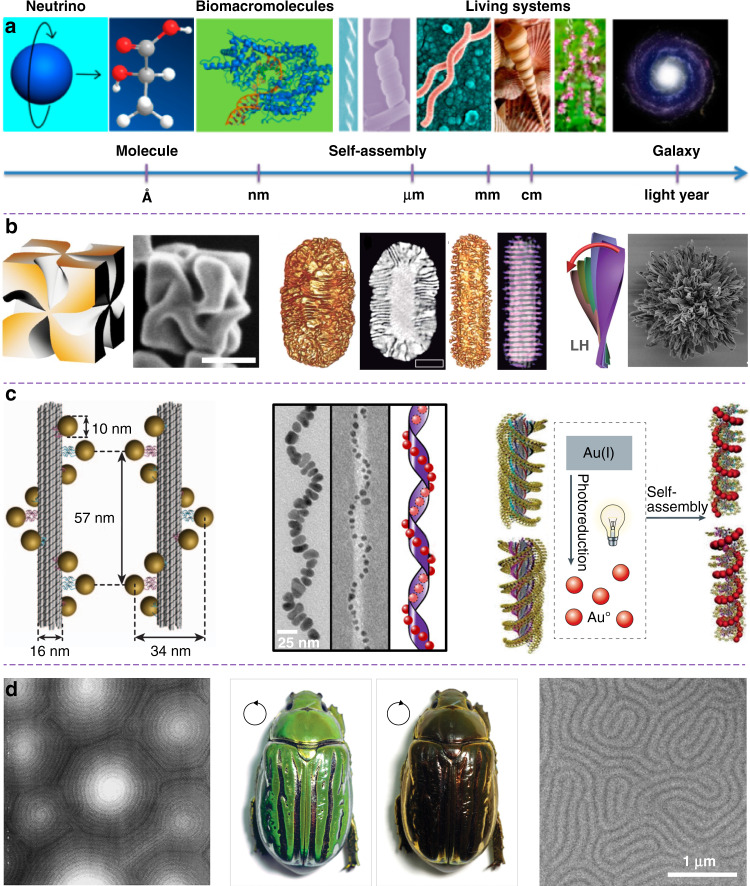
Chirality at the nanoscale. **a** Chirality at various hierarchical scales. Reproduced with permission from ref. ^2^. Copyright 2015, American Chemical Society. **b** Chiral nanomaterials with intrinsic chirality prepared by enantioselective synthesis. Left: Reproduced with permission from ref. ^17^. Copyright 2018, Springer Nature. Middle: Reproduced with permission from ref. ^21^. Copyright 2020, AAAS. Right: Reproduced with permission from ref. ^18^. Copyright 2020, AAAS. **c** Chiral nanomaterials based on different soft templates (left-to-right): DNA-, peptide-, and chiral gelator-directed self-assembly of achiral nanoparticles. Left: Reproduced with permission from ref. ^23^. Copyright 2012, Springer Nature. Middle: Reproduced with permission from ref. ^26^. Copyright 2016, American Chemical Society. Right: Reproduced with permission from ref. ^37^. Copyright 2014, American Chemical Society. **d** Chiral liquid-crystalline nanoarchitecture observed in beetles and biomimetic systems. (Left) Atomic force microscopy image of the beetle exoskeleton. (Middle) Beetle exoskeletons under left- and right-handed circularly polarized light. (Right) Scanning electron microscope image showing the self-assembly of platinum nanoparticles around the fingerprint of a cholesteric liquid-crystalline template. Left and middle: Reproduced with permission from ref. ^85^. Copyright 2009, AAAS. Right: Reproduced with permission from ref. ^89^. Copyright 2002, Springer Nature

Chirality is omnipresent in living organisms and nature. Chiral architectures can be found at a variety of hierarchical levels, ranging from atomic, molecular to supramolecular, macroscopic, and galactic scales (Fig. 1a)^1,2^. Chirality is known to be of paramount significance for multidisciplinary fields from biology, medicine, material science to high-energy physics. In biology, homochirality is believed to be a prerequisite for the genesis of life, however, the origin of biological homochirality, i.e., the selection of left-handed (_L_) amino acids and right-handed (_D_) sugars as molecular building blocks of life, has remained a great mystery since its discovery in the 19th century^3^. In medicine, chirality is a fundamental hallmark in drug development, evidenced by the fact that many of the drugs discovered are chiral. Interestingly, one enantiomer of chiral drugs may be highly effective for a particular disease, whereas the opposite enantiomer may be inactive and even toxic^4^. It should be noted that the chirality at a molecular scale is inherently weak, and extending the chirality from molecules to nanomaterials could bring many new opportunities for the design and synthesis of emerging chiral functional nanomaterials with a remarkable improvement in circular dichroism (CD) and polarization rotation, features that hold immense technological applications in sensing, imaging, medicine, catalysis, nonlinear optics and advanced electronics^5,6^. Chiral nanomaterials are expected to act as a powerful bridge platform for fundamental research of the chirality transfer and amplification between molecules and bulk materials since their physical and chemical properties can be facilely tailored by modulating their shape, size, charge, composition, and functional groups^7^. Recently, we have witnessed many outstanding achievements about synthesis, property, and application of chiral nanomaterials^8–11^, and great endeavors have been devoted to developing advanced chiral nanomaterials with marked enhancement of optical asymmetry, dynamic and tunable chirality, as well as unprecedented chiroptical activity in various specific wavelengths ranging from the ultraviolet, visible to near-infrared and terahertz regions^12–14^. There is no doubt that chiral functional nanomaterials are currently at the forefront of both fundamental research and technological applications.

Most nanomaterials are known to thermodynamically exhibit achiral crystal structures. To obtain chiral nanomaterials, researchers have developed two mainstream bottom-up strategies to introduce intrinsic chirality or spatial asymmetry into advanced functional nanostructures^15,16^. The most straightforward method is the enantioselective synthesis of chiral nanocrystals with mirror-asymmetric geometry via seed-mediated colloidal growth. Various chiral nanomaterials with a broken mirror and inversion symmetry have been successfully achieved using either chiral amino acids and peptides^17–20^ or chiral co-surfactant micelles^21^ as molecular modifiers (Fig. 1b). Interestingly, nanoscale chirality can also be realized from directed self-assembly of achiral functional nanoparticles using various chiral soft templates such as DNA^22–25^, peptide and protein^26,27^, liquid crystal (LC)^1,2,28,29^, chiral polymer^30–32^, and organogelators^33–37^ (Fig. 1c). Chiral nanomaterials based on emerging soft templates have been considered as the model system for investigating their inter-particle coupling, high-order self-organized nanostructures, and responsive dynamic properties, which can bring up a variety of potential applications^38–40^. For example, chiral nanomaterials based on nanoscale plasmonic building blocks, i.e., chiral plasmonic nanomaterials, have been demonstrated to show novel plasmon coupling or collective plasmonic properties that are usually absent in their discrete counterparts^41–47^. Chiral plasmonic nanomaterials could exhibit unprecedented enhanced CD, amplified dissymmetry factor (*g*-factor), engineerable and dynamic chiroptical responses^41^, thus holding a great opportunity in plasmon-based technological applications, such as chiral sensing^48,49^, chirality detection^50–52^, surface-enhanced spectroscopies^53,54^, and beyond^55,56^. By using novel chiral soft templates, nanoscale luminescent building blocks are able to self-organize into chiral luminescent nanomaterials exhibiting significantly enhanced circularly polarized luminescence^28,57–61^. Thanks to their promising photophysical properties, chiral luminescent nanomaterials have attracted significant attention in diverse areas, such as biological science^62^, 3D display^63^, spintronics^64,65^, enantioselective photochemistry^36,66^ and information encryption^67,68^.

Among the different chiral soft templates, liquid-crystalline soft templates are highly attractive for guiding the self-assembly of nanoscale functional building blocks into high-order chiral nanomaterials due to their inherent long-range ordered molecular assemblies that couple liquid fluidity with crystal ordering from molecular to macroscopic hierarchical levels^69–84^. As a matter of fact, chiral liquid-crystalline nanoarchitectures widely exist in many living organisms, such as in certain plant tissues, cuticles of insects and arthropods, including snail shells, beetle cuticles, butterfly wings, among others^85–88^. For example, the cuticle of jewel beetles *Chrysina gloriosa* exhibits selectively and circularly polarized iridescence due to the existence of chiral or cholesteric nanoarchitecture in their exoskeletons^85^ (Fig. 1d). Recently, many researchers have devoted themselves to the design and synthesis of advanced chiral functional nanomaterials using liquid-crystalline soft templates. Benefiting from chiral self-assembly feature of diverse chiral liquid-crystalline phases, such as 1D chiral nematics^89^, 2D chiral smectics, and 3D blue phases^90–104^, it is possible to transfer their chirality and periodicity into functional nanomaterials with unique and unprecedented functionalities. In this review, we present a comprehensive review of the state-of-the-art advances of liquid crystal-templated chiral nanomaterials and their promising applications. First, the critical chiroptical properties of chiral nanomaterials are introduced. Then, an overview of chiral functional nanomaterials is showcased, which includes chiral plasmonic nanomaterials based on thermotropic and lyotropic liquid crystal templates, as well as chiral luminescent nanomaterials based on different nanoscale building blocks, such as emerging inorganic quantum dots, perovskite nanocrystals, and upconversion nanoparticles. Finally, we conclude this review with an outlook on the potential scope, opportunities, and future challenges in emerging applications of these intriguing chiral functional nanomaterials. This review is expected to not only deepen the understanding of the fundamentals of soft-matter chirality, but also bring new twists in the development of advanced chiral functional nanomaterials and their promising applications in the fields of chiral plasmonics and transformational chiral photonics such as optical spintronics, quantum communication, optical information processing, and beyond.

## Chiroptical properties of chiral nanomaterials

**Fig. 2:**
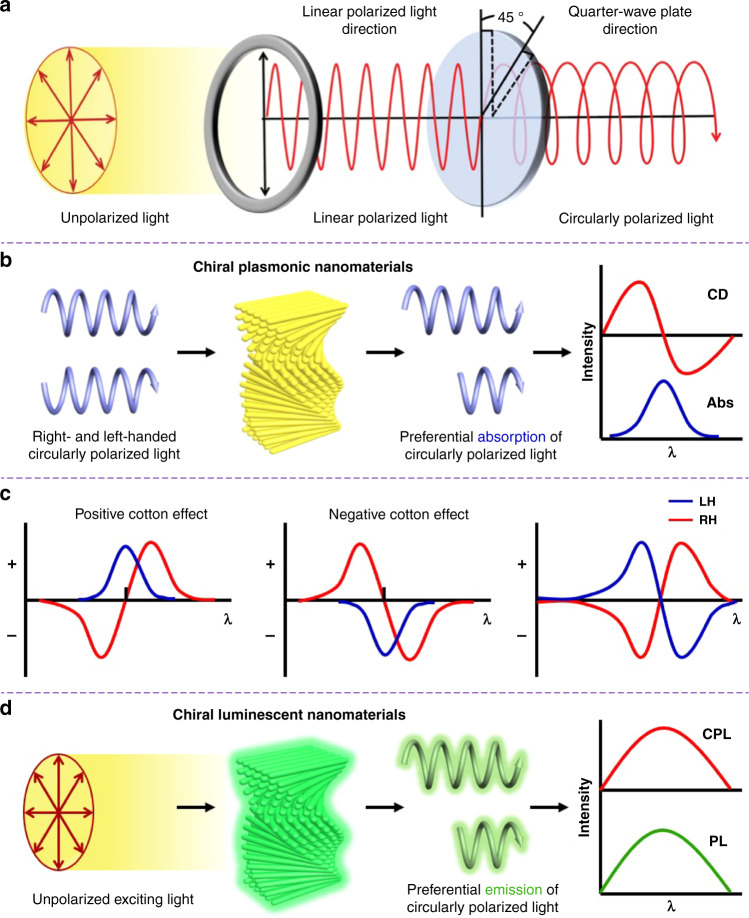
Chiroptical properties of chiral nanomaterials. **a** The relationship between unpolarized light, linearly polarized light and circularly polarized light. Reproduced with permission from ref. ^111^. Copyright 2020, WILEY-VCH. **b** Schematic illustration of circular dichroism (CD). **c** Typically cotton effect (left and middle) and bisignate CD spectra (right) in chiral nanomaterials. **d** Schematic illustration of circularly polarized luminescence (CPL)

The strong interaction of chiral nanomaterials with electromagnetic waves in the spectral regions, ranging from microwave and terahertz to infrared, visible and ultraviolet regions, is the fundamental basis for the generation of enhanced chiroptical activity and related optical spectroscopic characterization technologies^105–109^. Generally, the light coming from either sunlight or other light sources can be considered as unpolarized light, in which its electromagnetic waves, i.e., electric and magnetic fields, are emitted and randomly propagated at different polarization angles and varying rapidly in time. In contrast, polarized light can be described simply as a wave vibrating in a particular direction and, depending on the wave vector path perpendicular to the direction of propagation of the light wave, it can be classified as partially, elliptically, linearly, and circularly polarized light^110^. For instance, linearly polarized light has its electromagnetic fields, orthogonal to each other, but being the electric-field polarized along the x-axis. Circularly polarized light is a combination of two linearly polarized orthogonal waves of the same amplitude but with a phase difference of π/2, resulting from passing linearly polarized light through a quarter-wave plate; however, this process causes the loss of about 50% of light due to both the process of passing through the plates and the conversion of the unpolarized light into linearly polarized light (Fig. 2a). The handedness of circularly polarized light is determined by the rotational orientation of the vector of a wave approaching an observer, if the approaching light is rotating clockwisely, the light is right-hand circularly polarized light; otherwise, it is called left-hand circularly polarized light. It is worth noting that chiral nanomaterials can manifest themselves optically via a different response to right- or left-hand circularly polarized light.

Optical spectroscopy, such as CD spectroscopy that originates from the differential absorption of left-hand and right-hand circularly polarized light, has been considered as one of the most widely used techniques for the in-depth investigation of chiroptical properties of chiral functional nanomaterials^111,112^. For measurement, alternately left-hand and right-hand circularly polarized light generated from a xenon lamp is passed through optically active chiral nanomaterials. The CD spectrum is then obtained by comparing the intensity of the original and the remaining light (Fig. 2b). The observation of a peak or valley in the CD spectrum is known as the Cotton effect, which is the characteristic shift of the CD near an absorption band of a compound (Fig. 2c). Upon decreasing the wavelength, if the CD increases first, then the Cotton effect is positive, otherwise it is negative^2^. It is important to note that due to their proportionality, the oscillator strength (a positive quantity) and the rotational strength (a positive or negative quantity) determine the transition intensity in the absorption and CD spectra, respectively. Consequently, such optical signals are always positive in the absorption spectra, while positive or negative signals are found in the CD spectrum depending on the sign of the rotational force.

For chiral plasmonic nanomaterials, as shown in Fig. 2c, the resonant plasmonic coupling between individual nanoscale building blocks results in the appearance of bisignated CD signals in the spectral region of plasmon resonances, which is also known as exciton coupled circular dichroism (ECD)^105^. For right-handed chiral nanomaterials, a positive signal in the bisigned CD spectrum is often observed within the long-wavelength region and a negative signal observed in the short wavelength region. It should be noted that both signals are reversed for left-handed chiral nanomaterials. The dissymmetry factor (*g*-factor) is always used to measure polarization efficiency, so that we can exclude the influence of the concentration of sample and the path length of light. Specifically, the CD dissymmetric degree can be quantified using the absorptive dissymmetry factor (*g*_abs_, also known as *g*_CD_), a dimensionless quantity expressed as the difference between the absorbance of right-hand and left-hand circularly polarized light (A_L/R_) in comparison to the absorbance of nonpolarized light (A) at a given wavelength^105^:

1a $$g_{abs} \,=\, \frac{{A_L \,-\, A_R}}{A}$$

1b $$\,=\, \frac{{A_L \,-\, A_R}}{{(A_L \,+\, A_R)/2}} \,=\, \frac{{2\left( {A_L \,-\, A_R} \right)}}{{A_L \,+\, A_R}}$$

It is worth noting that in Eq. 1b, the absorbance in the denominator is expressed as the average of the absorbances of right-hand and left-hand circularly polarized light. However, both definitions for *g*_abs_ (Eqs. 1a, b) are employed indistinctly in the research^105^. Experimentally, the value of *g*_abs_ is defined as Eq. 2:

2 $$g_{abs} \,=\, \frac{{ellipticity \,\times\, absorbance}}{{32980}}$$

where the “ellipticity” (unit: mdeg) and the “absorbance” can be directly obtained from CD spectra. Besides the CD spectra, the chiroptical activity of chiral nanomaterials can also be measured by optical rotation dispersion (ORD) and vibrational circular dichroism (VCD)^5^. For CD and ORD, they are mathematically connected via the Kronig-Kramers equation. CD is based on the absorption difference when circularly polarized light is passed through a chiral matrix, while ORD arises from the scattering difference. The main difference of CD and VCD is that they work in different optical wavelength ranges. CD spectrum is located in the ultraviolet-visible region while VCD is located in the infrared region.

For chiral luminescent nanomaterials, there is another important chiroptical property, i.e., circularly polarized luminescence (CPL). Such nanomaterials upon being excited are capable of emitting light as they relax to the ground state through a process called luminescence. Analogously to CD spectroscopy, CPL spectroscopy is used to measure the difference between the intensity of light emitted with right-hand and left-hand circular polarization, as illustrated in Fig. 2d^111^. For measurement, unpolarized light is used to excite the sample, and the emitted light is passed through a circular analyzer composed of a photoelastic modulator and linear polarizers. The very-low-frequency photoelastic modulator alternately converts the right- or left-hand circularly polarized light into linearly polarized light, which then passes through the linear polarizers to finally reach the monochromator and detector. It is advisable to quantify the chiroptical property of an emissive chiral nanomaterial using the luminescence dissymmetry factor (*g*_lum_ or *g*_em_). In strict analogy with the *g*_abs_ in Eq. (1b), *g*_lum_ is expressed as the ratio of the difference between the intensity of right-hand and left-hand circularly polarized emission (*I*_*L*_ − *I*_*R*_) to the average intensity of emitted light at a given wavelength (Eq. 3)^111^:

3 $$g_{lum} \,=\, \frac{{2(I_L \,-\, I_R)}}{{I_L \,+\, I_R}}$$

It is very clear that Eq. 1b and Eq. 3 share a similar mathematical expression. Interestingly, the values for both CD and CPL vary between +2 to −2, with ±2 being the maximum value when there is an ideal right-hand or left-hand circular polarization of emitted light, whereas a zero value indicates the absence of CPL. It should be noted that chiral luminescent nanomaterials normally display the Cotton effect, but the CD signals do not ensure the observation of CPL signals. This can be attributed to the fact that the absorption always occurs from a state at thermal equilibrium, whereas luminescence occurs from an excited state. Therefore, the CPL spectrum elucidates both conformational and configurational properties of the excited state, whereas CD provides useful information of the ground state. In addition to optical spectroscopy, many morphological characterization technologies, such as transmission electron microscope (TEM), scanning electron microscope (SEM), atomic force microscope (AFM) and scanning tunneling microscope (STM) have been widely applied for direct observation or visualization of chiral functional nanomaterials^113^.

It should be noted that chiral plasmonic nanomaterials are known to exhibit strong CD but *g*_abs_ is very low. Most chiral nanomaterials strongly scatter the light and the *g*_abs_ is often between 10^−4^ and 10^−2^, which is much lower than that for chiral liquid crystal^114–116^. Recently, Liu et al. overcame this limitation by establishing the long-range self-assembly of plasmonic nanoparticles, similar to the liquid crystals. It was found that liquid crystal-like order could significantly convert low-*g*_abs_ plasmonic nanoparticles into high-*g*_abs_ chiral nanomaterials with an increase of more than 4600-fold^117^. Interestingly, chiral liquid-crystalline templates have been widely applied for effectively amplifying the *g*_lum_ of chiral luminescent nanomaterials^28^. Basically, cholesteric or chiral nematic liquid crystals (CLCs) with helical nanostructures are the most common method used for *g*_lum_ amplification with chiral liquid-crystalline templates. One of the features that makes CLCs the most attractive chiral templates is the photonic bandgap effect, that is, the selective reflection of circularly polarized light with the same handedness while the transmission of the opposite one. This is particularly important for generating large *g*_lum_ from liquid crystal-templated chiral nanomaterials^118^. When the luminescence peak of chiral nanomaterials does not overlap with the reflection band of CLCs, the optical rotation from CLCs results in a high degree of CPL polarization, and the polarized direction of CPL is mainly determined by the handedness of CLCs. When the luminescence peak of chiral nanomaterials coincides with the reflection band of CLCs, the CPL with the same direction as the helical twist of CLCs is fully reflected, whereas the CPL with the opposite handedness is fully transmitted. For example, Shi et al. conducted experimental and theoretical studies on the emission outside the reflection band of liquid-crystalline templates, and it was found that CLC-templated fluorescent films displayed CPL with *g*_lum_ about 0.8^119^. Chen et al. reported that almost pure CPL (*g*_lum_ = ∼2) was observed for CLC-templated fluorescent films when the emission occurred inside the reflection band^120^. Hence, a significantly enhanced *g*_lum_ value is expected to be obtained by localizing the luminescence of chiral nanomaterials at the center of the photonic bandgap of CLCs^121,122^. However, the loss of at least half of the intensity of the CPL could be an unavoidable issue. To solve the energy loss issue of CLC templates, the development of cholesteric films with hyper-reflection as chiral liquid-crystalline templates will be an effective strategy to obtain CPL with large *g*_lum_ and high emission intensity. In nature, the beetle *Plusiotis resplendens* is known to exhibit hyper-reflection that breaks through the typical reflectance limit of 50% due to its unique three-layer superstructure, in which a unidirectional layer acting as a half-wave retardation plate is embedded between two layers of left-handed cholesteric layers^123^. Several strategies have been proposed for the development of chiral liquid crystals with hyper-reflection^124,125^. For example, Matranga et al. fabricated a biomimetic hyper-reflective liquid-crystalline film by sandwiching an untwisted nematic layer, acting as a half-wave retarder, between multiple cholesteric layers^126^. This multilayering method is expected to prepare chiral liquid-crystalline templates capable of reflecting both left- and right-handed circularly polarized light. Mitov and Dessaud demonstrated that by introducing a temperature-sensitive chiral molecular switch and controlling the photopolymerization temperature, hyper-reflection can be achieved in a single-layered cholesteric film with left- and right-handed cholesteric nanostructures^127^. Moreover, a “washout/refill” strategy has been developed to fabricate the single-layered chiral liquid-crystalline templates with hyper-reflection^128,129^. It is expected that the application of promising multi-layered or single-layered chiral liquid-crystalline templates with hyper-reflection can be an effective strategy for obtaining CPL with large *g*_lum_ and high emission intensity.

## Liquid crystal-templated chiral plasmonic nanomaterials

Chiral plasmonic nanomaterials with giant localized surface plasmon resonance (LSPR) have received increasingly significant attention for their emerging potential applications in diverse fields, such as negative-refractive-index materials, ultrasensitive biosensing, enantioselective analysis, and advanced light-polarization filters^130–138^. Compared to conventional top-down fabrication strategy^139–142^, the bottom-up self-assembly approach based on chiral soft templates allows for arbitrary and more tailorable structural geometries, thus enabling the development of highly complex chiral plasmonic nanomaterials with engineerable and dynamic chiroptical responses^41^. In 1996, Burkett and Mann pioneered biolipid-templated chiral nanomaterials, in which the helical edges of lipid ribbons were used as chiral templating patterns to transcribe a roughly helical arrangement of gold nanoparticles^143^. In 2009, Sharma et al. first reported DNA-templated chiral nanomaterials through the co-assembly of DNA and oligonucleotide functionalized gold nanoparticles into 3D tubules, demonstrating the versatility of DNA-based soft templates and opening the proverbial floodgates to the deep exploration in this area^144^. In 2012, Kuzyk et al. demonstrated a DNA origami chiral soft template that could offer nine helically-arranged attachment sites for oligonucleotide-modified plasmonic nanoparticles, which led to the development of high fidelity chiral nanomaterials with characteristic bisignated CD signals^23^. Peptide-based chiral soft templates have been developed for fabricating diverse chiral plasmonic nanomaterials with tailorable chiroptical properties^26,145–147^. Different from other chiral soft templates, chiral liquid-crystalline templates are known to show multitudinous advantageous attributes, such as long-range molecular ordering, structural diversity of chiral mesogenic phases, and superior responsiveness to many external stimuli, such as light, temperature, electric field, mechanical force^1,2,77,98^. Importantly, many researchers have demonstrated that using chiral liquid crystals as soft templates can effectively amplify the chirality of nanomaterials and increase the optical asymmetry^148–151^. In this section, we shall introduce recent endeavors in the development of chiral plasmonic nanomaterials with various thermotropic and lyotropic liquid crystal templates.

### Chiral nanomaterials based on thermotropic liquid crystal templates

**Fig. 3:**
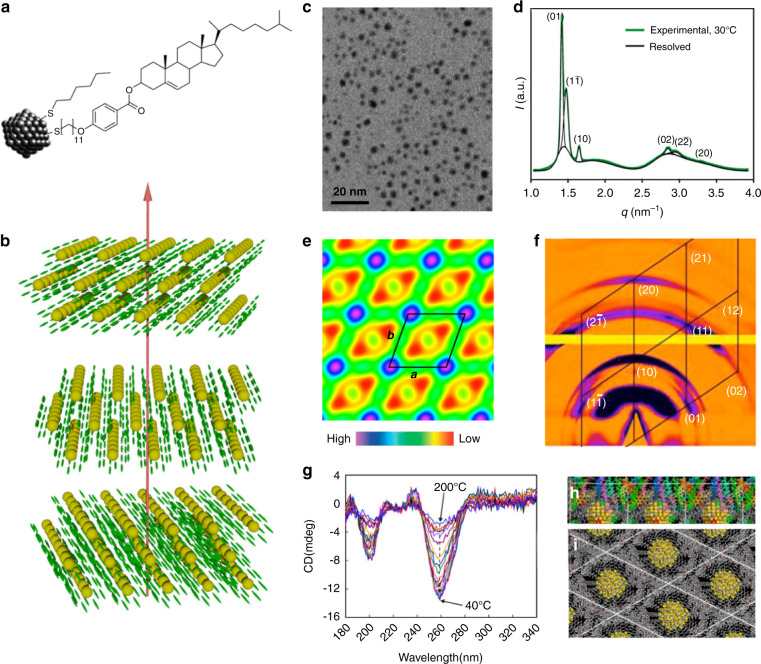
Self-assembled chiral plasmonic nanomaterials made from mesogenic ligands-capped gold nanoparticles. **a** Schematic structure of mesogen-functionalized gold nanoparticle AuCholC6. **b** Schematic representation of the chiral plasmonic nanomaterials exhibiting chiral columnar liquid-crystalline phase. **c** TEM image of AuCholC6. **d** SAXS curve of annealed AuCholC6. **e** Electron density mapping of the columns based on the SAXS reflections. **f** GI-SAXS pattern of a slowly cooled AuCholC6 nanostructured film in the formed mesophase. **g** CD spectra of a AuCholC6 nanostructured film upon cooling from the isotropic-liquid-crystalline phase at 200 °C to 40 °C. **h**–**i** Side and top views of modeling AuCholC6 nanostructures based on molecular dynamics simulation. Reproduced with permission from ref. ^167^. Copyright 2015, American Chemical Society

Thermotropic liquid crystals are known to exhibit different mesophases upon changing the temperature within a certain range. They are made up of mesogenic compounds exhibiting diverse molecular geometries, such as rod-like (calamitic), disk-like (discotic), bowl-like (bowlic), bent-shaped or banana-shaped structures^152–155^. Generally, the development of single-component chiral mesogens and the construction of host-guest material systems have been the two most widely adopted methods for introducing chirality into thermotropic liquid crystals. The latter method is accomplished by the addition of appropriate mesogenic or non-mesogenic chiral dopants into an achiral liquid-crystalline host. Chiral thermotropic liquid crystals could show a variety of mesophases, such as CLCs with helicoidal organization, chiral smectic with spontaneous polarization, twist grain boundary (TGB) phase with frustrated helical superstructures, and blue phases with cubic nanostructures^156,157^. CLCs are one of the most commonly used thermotropic liquid-crystalline templates, which can serve as surface ligands or host matrices to guide the self-assembly of discrete and nanoscale plasmonic building blocks into chiral plasmonic nanomaterials. In 2002, Mitov et al. obtained long-range ordered chiral nanomaterials by doping platinum nanoparticles into a CLC host matrix^89^. They found that the dispersed nanoparticles were self-organized into well-defined ribbons that mimicked the fingerprint pattern of the CLC template because they showed an affinity for regions where the liquid crystal molecules were parallel to the plane of the film^158^. The distance between the ribbons was found to highly depend on the chirality of the CLC template and could be facilely controlled by adjusting the helical pitch. In recent years, many researchers have devoted extensive efforts to the design and synthesis of chiral plasmonic nanomaterials through capping plasmonic nanoparticles with mesogenic ligands and controlling their bottom-up self-assembly process^159–166^. For example, Cseh et al. obtained chiral plasmonic nanomaterials from the gold nanoparticles coated with cholesterol-based chiral mesogenic ligands without the addition of a separate liquid-crystalline matrix (Fig. 3a)^167^. The resulting chiral plasmonic nanomaterials were found to exhibit a chiral columnar liquid-crystalline phase owing to the cholesteric arrangement of the mesogens with threaded gold nanoparticle strands (Fig. 3b). The layers of gold columns superimposed at a small twist angle to each other coiled around the vertical axis forming a helix. Upon annealing, a columnar liquid-crystalline nanostructure was gradually formed with the nanoparticles forming strands in a regular oblique 2*d* lattice as confirmed by grazing-incidence small-angle X-ray scattering (GI-SAXS), and a significant increase of the synchrotron radiation CD signal at the isotropic-to-liquid-crystalline phase transition temperature was also observed (Fig. 3c–i). The disclosed strategy opened a new direction for the design of plasmonic metamaterials or chiral plasmonic nanomaterials that can selectively interact with circularly polarized light. Similarly, Yu et al. reported the preparation of chiral plasmonic nanomaterials using chiral discogen ligands-functionalized gold nanoparticles, which formed a stable chiral discotic nematic phase in the liquid-crystalline state^168^. Bhat et al. directly synthesized mesogenic ligands-encapsulated chiral gold nanoparticles by in situ reductions of Au(III) to Au(0), and demonstrated their self-assembling into a fluid/frozen chiral nanomaterials exhibiting a strong CD activity^169^. By attaching photoactive chiral mesogenic ligands to the surface of gold nanoparticles, Bhardwaj et al. reported photoresponsive chiral plasmonic nanomaterials exhibiting dynamic regulation of electromagnetic response^170^. The resulting chiral nanomaterials were found to exhibit tunable epsilon-near-zero behavior with a bandwidth of ∼45 nm in the visible spectrum, which can be enhanced by a factor of 1.6 upon UV illumination. The concept of sergeant-soldier rule^171–173^, which means that large volumes of achiral molecules (regarded as soldiers) obey the conformation of small amounts of chiral dopants (regarded as sergeants) to afford helical torsion, has been considered an effective approach to amplify supramolecular chirality. It was also found that the LSPR effect of plasmonic nanomaterials can be utilized for significantly amplifying the chirality of surrounding chiral species^174–176^. Mesogen-functionalized chiral plasmonic nanoparticles can directly function as chiral nanodopants to induce the formation of chiral nematic liquid crystals. The chiral plasmonic nanomaterials could also exhibit larger chiral correlation lengths, long-range interactions between chiral molecules and plasmonic nanomaterials, and enhanced *g*-factor for chiral molecules in the vicinity of plasmonic nanomaterials^177–179^. Interestingly, an array biconvex converging microlens with robust imaging capabilities has been fabricated using liquid crystal-templated chiral plasmonic nanomaterials^180^.

**Fig. 4:**
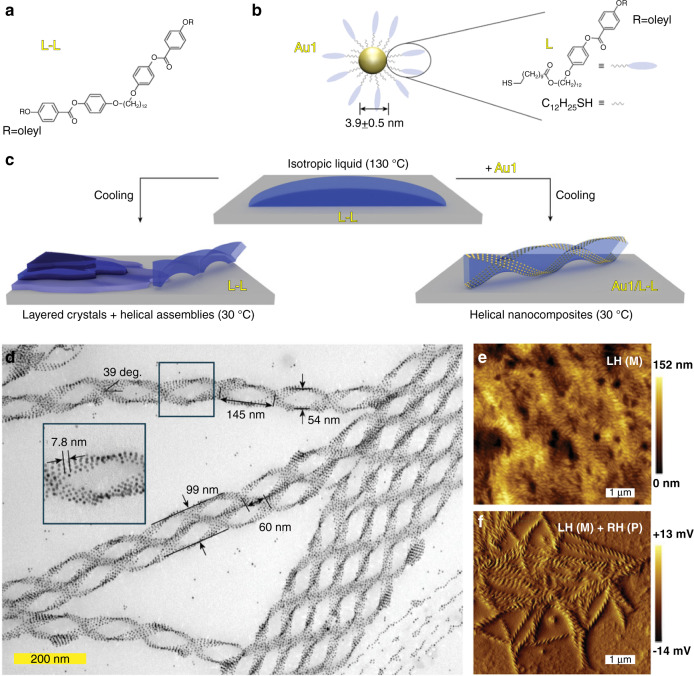
Self-assembly of mesogen-functionalized gold nanoparticles into left- and right-handed chiral nanomaterials with thermotropic liquid-crystalline template. **a** Molecular structure of a mesogenic dimer (L–L). **b** Schematic structure of the mesogen-functionalized gold nanoparticle. **c** Schematic self-assembly process of chiral plasmonic nanomaterials. **d** TEM image of chiral nanomaterials. **e**, **f** AFM images of left- and right-handed chiral nanomaterials. Reproduced with permission from ref. ^188^. Copyright 2020, WILEY-VCH

Recently, emerging bent-core mesogens exhibiting the B4 phase or so-called helical nanofilament (HNF) phase have been used as chiral liquid-crystalline templates for fabricating chiral plasmonic nanomaterials^44,159,181–187^. For example, Lewandowski et al. judiciously designed and synthesized a dimeric liquid-crystalline template (L–L), which can self-organize into the HNF phase showing hierarchical and tailorable nanostructures and drive the self-assembly of L-ligand-functionalized gold nanoparticles into ordered chiral plasmonic nanomaterials^188^. The presence of plasmonic nanoparticles did not affect the nucleation and growth processes of the HNFs, so they ended up located at the lateral boundaries of the structure, thus stabilizing the air-HNF assembly (Fig. 4a–c). The resulting chiral nanomaterials showed outstanding long-range hierarchical order across length scales and their thickness could be reversibly modulated thanks to the dynamic and reconfigurable nature of liquid-crystalline materials (Fig. 4d). The self-assembly process can be facilely controlled by changing the cooling rate from the isotropic phase, and adjusting the molar ratio between the nanoparticles and LC matrix. It should be noted that a spontaneous symmetry breaking process can be observed in the HNF phases, because at the nucleation stage the formed dendritic domains do not show preferential handedness, i.e., left-handed and right-handed helical nanomaterials are obtained with equal probability, resulting in racemic samples at bulk scale (Fig. 4e, f).

**Fig. 5:**
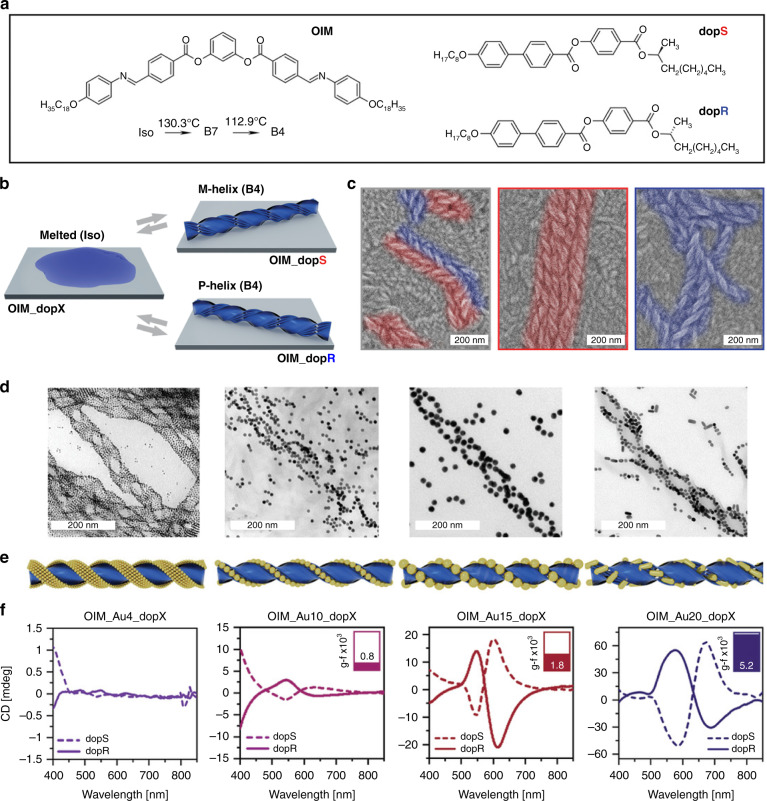
Thermotropic liquid crystal-templated chiral plasmonic nanomaterials. **a** Molecular structures of liquid crystal matrix (OIM) and chiral dopants (dopS and dopR). **b** Schematic formation of left-handed (M-type) and right-handed (P-type) chiral nanomaterials. **c** SEM images showing M- and P-helices formed in pure liquid crystal matrix, dopR-doped liquid crystal and dopS-doped liquid crystal, respectively; P-helices and M-helices are colored in blue and red, respectively. **d**–**f** TEM images, 3D models, and corresponding CD spectra of chiral nanomaterials prepared using 4, 10, and 15 nm spherical gold nanoparticles, and 8 × 20 nm gold nanorods. Reproduced with permission from ref. ^190^. Copyright 2022, WILEY-VCH

Interestingly, the identification and mechanical removal of domains formed in homochiral nanomaterials allow the measurement of plasmonic CD signals from microscale regions^189^. By using micro-CD measurements, Szustakiewicz et al. could directly identify the handedness of specific domains based on the sign of the ΔExt, where the ΔExt indicates the difference between extinction of right-handed and left-handed circularly polarized light. CD signals with a visible Cotton effect were observed around the LSPR absorbance peak upon selective removal of domains with the same handedness. To control the chirality on a bulk scale, Grzelak et al. fabricated a centimeter-scale thin film of chiral nanomaterials with plasmonic CD by temperature-driven self-assembly of a mixture composed of a liquid-crystalline matrix, a chiral dopant, and gold nanoparticles^190^. The strategy proved to efficiently select chirality by allowing the chiral dopant to act in the nucleation phase and provide an enantiomeric excess of HNFs of a given handedness in the thin film (Fig. 5a–c). A series of experimental parameters, such as size, content and morphology of nanoparticles and amount of chiral dopant, were systematically investigated to optimize chiral plasmonic films. Interestingly, double-helical assemblies of gold nanoparticles were observed in the resulting chiral plasmonic thin films (Fig. 5d). Helically twisted 1D chains were obtained in large spherical nanoparticles named as Au10 and Au15 (10 and 15 nm in diameter, respectively), while small Au4 NP formed helically twisted 2D ribbons. In contrast, the Au20 NRs (8 × 20 nm) were found to be preferentially coupled side-by-side in the nanostructured plasmonic film (Fig. 5e). The size of gold nanoparticles was shown to be positively correlated with the plasmonic CD intensity (Fig. 5f), and variation of the film temperature could allow reversible switching of the chiroptical response. Moreover, stretchable chiral plasmonic films were fabricated through thermal nanoimprinting and transfer-printing of the films onto a flexible substrate, and the resulting flexible film was found to exhibit a decreasing CD strength upon stretching and a recoverable chiroptical response upon relaxation.

### Chiral nanomaterials based on lyotropic liquid crystal templates

**Fig. 6:**
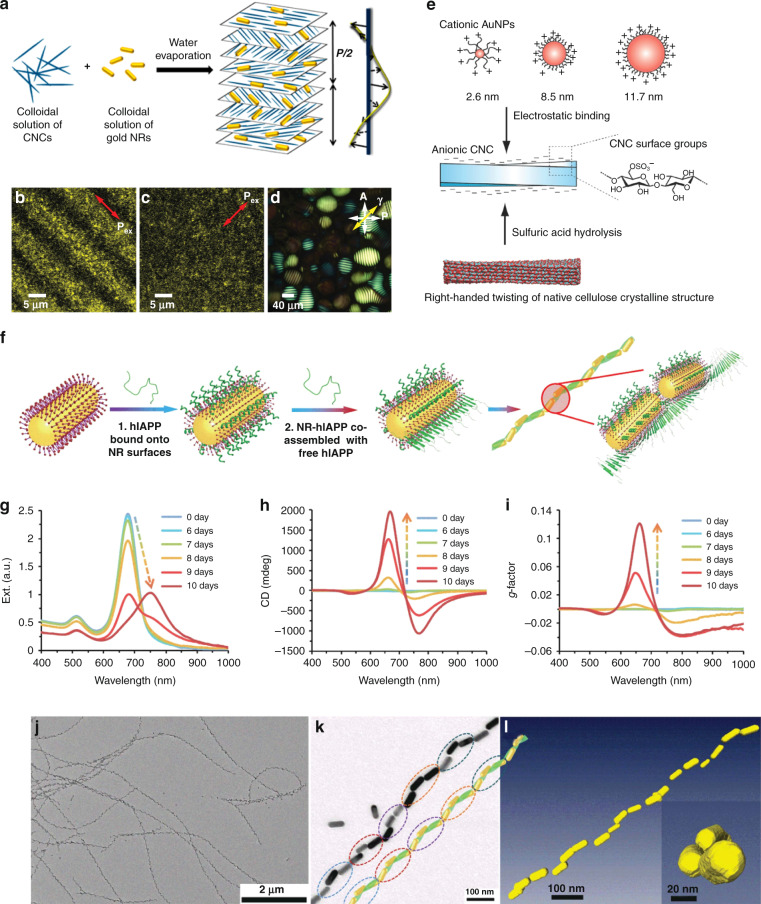
Lyotropic liquid crystal-templated chiral plasmonic nanomaterials. **a** Schematic chiral nematic ordering of gold nanorods based on CNC soft template. Reproduced with permission from ref. ^206^. Copyright 2014, American Chemical Society. **b–d** Helicoidal assembly of gold nanorods in colloidal CNC template exhibiting polarization-dependent plasmonic extinction. Reproduced with permission from ref. ^210^. Copyright 2014, WILEY-VCH. **e** Chiral plasmonic nanomaterials based on the electrostatic interactions between CNCs and plasmonic gold nanoparticles. Reproduced with permission from ref. ^214^. Copyright 2016, WILEY-VCH. **f–l** Peptide assembly-enabled liquid crystal-like plasmonic helices with long-range order. Reproduced with permission from ref. ^117^. Copyright 2021, AAAS. **f** Schematics self-assembly process of hIAPP template and gold nanorods. **g** Extinction, (**h**) CD, and (**i**) *g*-factor spectra in the co-assembly process. **j**, **k** TEM images of nanohelices. **l** Reconstructed cryo-TEM tomography images of the left-handed helices

Lyotropic liquid crystals are known to exhibit diverse mesophases such as nematic, lamellar, cholesteric, cubic, and hexagonal columnar phases, as a function of the concentration of anisotropic suspensions with amphiphilic compounds or colloidal nanoparticles at appropriate solvents^1,2,191–197^. The formation of lyotropic liquid-crystalline phases has been observed in many material systems such as lipid, tobacco mosaic virus (TMV), deoxyribonucleic acid (DNA), and cellulose nanocrystals (CNCs)^198–201^, which could function as chiral soft templates to guide the self-assembly of plasmonic nanoparticles into optically active nanomaterials. For example, CNCs have received extensive attention due to its renewable, nontoxic, and low-cost properties^197^. CNCs are known as negatively charged, highly crystalline, high-aspect-ratio rod-like nanocrystals that are able to self-organize into a chiral nematic liquid-crystalline phase in sufficiently concentrated aqueous suspensions^202^. The helical organization can be preserved in free-standing solid CNC films through an evaporation-induced self-assembly (EISA) process, which makes CNCs an attractive chiral soft templating matrix compared to other lyotropic systems in solution environments and suitable for large-scale production^203–205^. Kumacheva et al. reported chiral plasmonic films via introducing plasmonic gold nanorods into a CNC-based cholesteric liquid-crystalline template^206^ (Fig. 6a). The resulting chiral plasmonic films not only preserved the cholesteric ordering of the CNC template but also retained the plasmonic resonance of gold nanoparticles, resulting in strong resonant plasmonic-photonic coupling and distinctive plasmon-induced chiroptical activity^206–209^. The CD responses of CNC-templated chiral plasmonic films could be tuned by changing the fabrication conditions such as size, surface charge and concentration of plasmonic components as well as helical pitch of the CNC template. Lukach et al. demonstrated that the concentration of plasmonic nanoparticle components exhibited a significant influence on the chiroptical properties of CNC-templated chiral plasmonic films compared to size and surface charges^208^. At sufficiently high concentration of plasmonic components in the films, a splitting in the CD signal was observed with the minima coinciding with the spectral position of extinction plasmonic peaks. Smalyukh’s research group demonstrated that the anisotropic plasmonic nanoparticles were uniaxially aligned with their long axes parallel to the local director of the liquid-crystalline domains of CNC-based chiral soft template, and polarization-dependent LSPR effect was observed (Fig. 6b–d)^210,211^. Chu et al. demonstrated CNCs-templated hybrid chiral nematic films by the coalignment of anisotropic plasmonic silver nanowires and CNCs host matrix, in which tunable chiroptical properties and a maximum *g*-factor of 0.108 has been achieved^212^. It was found that the CD signals of the hybrid films could be modulated by changing the electrostatic repulsions between CNCs and slender silver nanowires. To shorten the processing time (4–6 days) and improve color uniformity of CNC-based chiral soft template, Feng et al. found that the introduction of a surfactant in CNC matrix could greatly enhance the orientation of multidomains in CNCs, and the existent surfactant was beneficial to increase the miscibility of plasmonic components and CNCs, resulting in chiral plasmonic films with uniform color and plasmonic chiroptical property^213^. Majoinen et al. reported that using individual CNC nanorods as soft template could also allow plasmonic chiroptical activity in dilute aqueous dispersions at nano/colloidal scale^214^. The nanoscale fibrillar chiral plasmonic nanomaterials were formed by electrostatically bonding the plasmonic components to individual CNC nanorods, and thus resulting in a marked chiral right-handed plasmonic CD signal. It should be noted that the handedness of CD signal was opposite to that of left-handed plasmonics of their liquid-crystalline assemblies due to inherent right-handed twist along CNC nanorod axis (Fig. 6e). Moreover, mesoporous CNC films^215^ or CNC-templated silica films^216,217^ were also used as chiral templates for fabricating plasmonic nanomaterials with intense chiroptical activity.

In addition to the widely used CNC-based chiral soft templates, amyloid fibrils, which are known as twisted and helical nanofibers from the self-assembly of β-sheet aggregates^218^, were also used as chiral liquid-crystalline templates. The self-assembly of amyloid fibrils is intimately associated with their orientation and ordering, which can be easily tailored by changing the concentration of aqueous amyloid dispersions^219–221^. For example, Marzán et al. used amyloid fibrils as chiral soft templates to prepare chiral plasmonic nanomaterials^179,222^, where the dispersed nanoscale plasmonic building blocks were guided into a chiral conformation, and a strong plasmonic CD response was obtained. Recently, Liu et al. reported human islet amyloid polypeptides (hIAPPs)-templated chiral plasmonic nanomaterials exhibiting significantly enhanced optical asymmetry *g*-factors^117^. By mimicking the long-range ordering of chiral liquid-crystalline molecules, high optical asymmetry at the nanoscale level was achieved with the self-assembly of gold nanorods into long helical chain-like nanorods after being conjugated to hIAPPs (Fig. 6f). Plasmonic CD signals of the bisignated line shape were observed upon nanohelix peptides-directed self-assembly of plasmonic building blocks. The intensity of the CD spectra was found to be as high as 2000 millidegrees (Fig. 6g, h), and the optical asymmetry *g*-factor of the chiral plasmonic nanomaterials reached a value of up to 0.12 (Fig. 6i), which was found to be more than 4600 times higher than that of the monomer. The plasmonic building blocks assembled into a long-range ordered chain with end-to-end orientation and left-handed nanohelices (Fig. 6j, k). The chiral nanomaterials were straight and up to 50 plasmonic building blocks were in registry with each other (Fig. 6l). The chiral response could be tuned by adjusting the nanorod’s size and the helix pitch. This work shines new light on the development of liquid crystal-templated chiral plasmonic nanomaterials and their emerging applications in complex biological media.

## Liquid crystal-templated chiral luminescent nanomaterials

Chiral luminescent nanomaterials exhibiting strong CPL are of overarching significance from the perspective of fundamental research and many technological applications since the circular polarization can function as a carrier of chemical or biological information for interdisciplinary fields such as biology, optics, photonics, spintronics optoelectronics, polarization-based information encryption, and related fields^223–225^. The conventional chiral luminescent nanomaterials through capping chiral ligands onto the surface of nanoparticles often exhibit a low luminescence efficiency and a low-*g*_lum_ value within the range of 10^−4^–10^−3^ ^226–228^. To address this problem, many alternative bottom-up self-assembly strategies have been developed for efficient amplification of nanoscale chirality in luminescent nanomaterials^229–231^. For instance, Tang et al. demonstrated supramolecular self‐assembly of chiral gold clusters with CD but free of CPL in crystalline nanocubes, and the resulting chiral nanomaterials were found to exhibit remarkably amplified CD intensity along with the appearance of the CPL signal^232^. Huo et al. reported white CPL-active chiral luminescent nanomaterials by supermolecular co-assembly of achiral quantum dots and chiral gelators^33^. Although the *g*_lum_ value could be increased by about an order of magnitude through the supramolecular self‐assembly approach, the values remain very low and far from the theoretical perfect value. Interestingly, chiral liquid-crystalline templates were found to be capable of effectively amplifying the *g*_lum_ of chiral luminescent nanomaterials^66,233,234^. Recently, a variety of emerging nanoscale building blocks such as inorganic quantum dots, perovskite nanocrystals, and upconversion nanoparticles have been applied for the development of liquid crystal-templated chiral luminescent nanomaterials exhibiting significantly enhanced circularly polarized luminescence. In this section, advanced chiral luminescent nanomaterials based on chiral liquid-crystalline templates are mainly introduced.

### Chiral quantum dots

Chiral quantum dots (QDs) combine the luminescent properties of quantum dots with the chiroptical activity of chiral nanomaterials, which can broaden their applicability to enantiomeric recognition and separation, asymmetric catalysis, bioimaging, and luminescence sensing^235^. Many researchers have devoted themselves to the design, synthesis, and properties of chiral quantum dots^236–238^. In 2013, Chiral ligand-induced chiroptical activity was observed in originally achiral semiconductor quantum dots^239^. Suzuki et al. demonstrated the generation of chirality on graphene quantum dots covalently functionalized with L/D-cysteine moieties^240^. Deka et al. designed and synthesized the fluorescent chiral carbon dots by incomplete carbonization of different chiral precursor molecules^241^. Li et al. synthesized the chiral carbon dots by the hydrothermal treatment of L- or D-cysteine, which can mimic topoisomerase I to mediate topological rearrangement of supercoiled DNA with enantioselectivity^242,243^. It should be noted that capping or covalent modification with other chiral molecules are the main strategies to introduce chirality in quantum dots. However, many challenging issues still exist such as complex synthesis procedures and weak CPL signals with *g*_lum_ values in the range of approximately 10^−3^–10^−2^ ^227^.

**Fig. 7:**
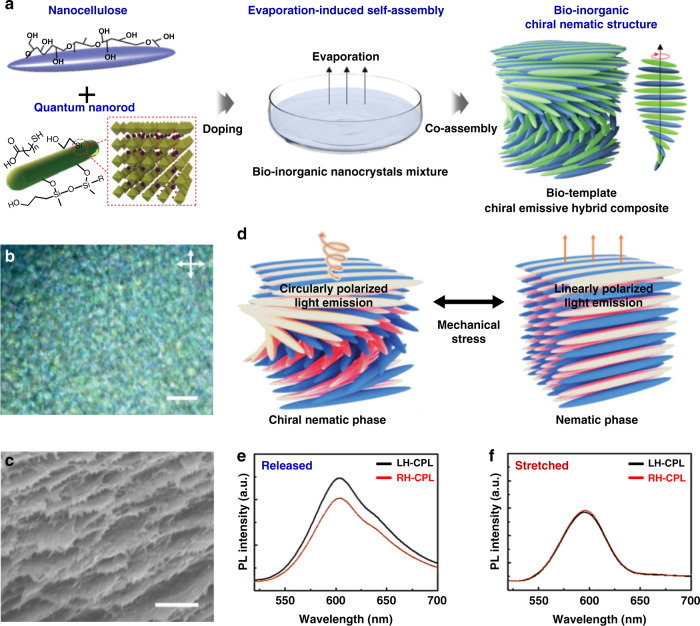
Chiral luminescent nanomaterials prepared from the co-assembly of CNCs and quantum nanorods. **a** Schematic process of fabricating chiral luminescent nanostructured film via evaporation-induced self-assembly. **b** Polarized optical microscope image of the chiral luminescent nanostructured film (Scale bar, 10 µm). **c** Chiral luminescent nanostructured films exhibiting chiral nanostructures. (Scale bar, 1 µm). **d–f** Mechanically driven polarization modulation of emitted light from CPL to linearly polarized emission. Reproduced with permission from ref. ^250^. Copyright 2021, WILEY-VCH

Recently, the use of chiral liquid crystals as soft templates to endow quantum dots with chiroptical activity has become a new research focus. Among various quantum dots, semiconductor quantum dots are widely used as nanoscale luminescent components to be incorporated into chiral liquid-crystalline templates due to their high quantum yield, tunable emission wavelengths, and good photophysical stability. For example, chiral luminescent nanomaterials have been reported through enclosing achiral semiconducting quantum dots in chiral liquid-crystalline templates, and the resulting chiral nanomaterials showed optically or electrically controlled CPL via conformational changes in the helical structure of chiral liquid-crystalline templates^244–246^. Shi et al. reported chiral luminescent nanomaterials by doping semiconductor quantum rods (QRs) into CNC-based chiral liquid-crystalline template^247^. The optical activity could be manipulated by regulating the relative position between the luminescent band of quantum rods and the photonic bandgap of CNCs, and reaching a maximum *g*_lum_ value of -0.45 when the luminescent band was located at the center of the photonic bandgap. Xu et al. demonstrated optical coding labels upon the introduction of semiconductor quantum dots into photonic CNC templates^68^. Thanks to left-handed 1D photonic nanostructures, iridescent photonic CNC templates with strong CD were able to transmit right-handed circularly polarized light and reflect left-handed circularly polarized light^248,249^, resulting in right-handed CPL. As a result, the code could be observed through a right-handed circular polarizer compared to a left-handed one. Interestingly, stretchable CNC template can be utilized to develop dynamic chiroptical systems that enables dynamic tuning of CPL by spatially changing the pitch or orientation when subjected to external stimuli^250–252^. For example, Kang et al. developed a novel chiral luminescent nanostructured film with dynamically reversible chiroptical properties by crosslinking chiral co-assembly of semiconductor quantum nanorods (QNRs) and CNCs into an elastomeric polyurethane matrix (Fig. 7a–c)^250^. The resulting chiral luminescent nanostructured film exhibited vivid structural color and generated strong CPL with *g*_lum_ of 0.2. It is worth noting that the asymmetric circularly polarized luminescence could be converted to linearly polarized light emission thanks to the mechanically induced transformation from a cholesteric helical superstructure to a unidirectional nematic nanostructure when the film was stretched (Fig. 7d–f). In addition, by introducing functional quantum dots into CNC-templated chiral nematic silica, free-standing chiral mesoporous films with simultaneous iridescence and luminescence were also demonstrated, and such luminescent mesoporous material could be used to detect traces of TNT explosives^253^.

**Fig. 8:**
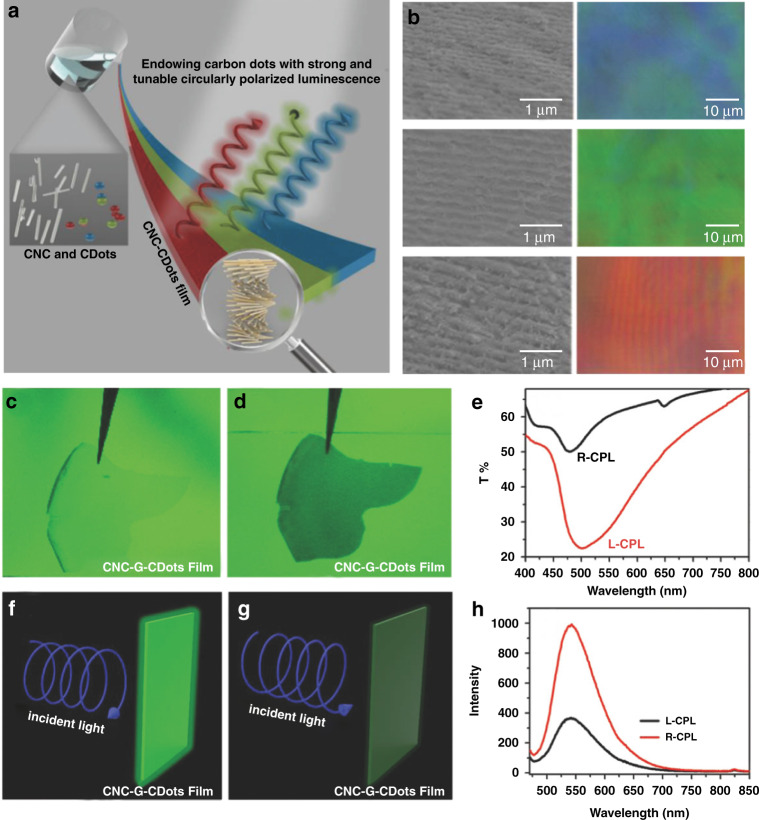
Chiral luminescent nanomaterials based on liquid-crystalline CNCs. **a** Schematic evaporation-induced co-assembly of carbon dots and CNC. **b** SEM images and polarized optical microscope images of chiral nanomaterials. **c**, **d** Transparent film and dark film upon exposure to right- and left-handed circularly polarized light, respectively. **e** Transmission spectra with left-handed and right-handed circularly polarized light. **f–h** Schematic representation of detecting circularly polarized light. Reproduced with permission from ref. ^257^. Copyright 2018, WILEY-VCH

Fluorescent carbon quantum dots share the unique electronic and optical characteristics of semiconductor quantum dots and offer great advantages over the existing limitations such as elemental shortages and/or intrinsic toxicity due to heavy metal use in the production of semiconductor quantum dots^254–256^. Many efforts have been devoted to the development of liquid crystal-templated chiral luminescent carbon quantum dots. For example, Zheng et al. fabricated circularly polarized luminescent nanostructured films via evaporation-induced cooperative assembly of carbon dots and CNCs suspensions^257^. By tuning the photonic bandgap of CNCs and the emission band of carbon dots, the resulting luminescent nanostructured films exhibited strong and multicolor-tunable right-handed CPL with *g*_lum_ up to −0.74 (Fig. 8a, b). It was found that superior photoemission intensity can be achieved when illuminating with right-handed circularly polarized light compared to left-handed circularly polarized light, thanks to the selective transmission of right-handed circularly polarized light and the low attenuation imparted by the left-handed helical superstructures of CNCs (Fig. 8c–h). Xu et al. observed both CPL and circular polarized room-temperature phosphorescence (CPRTP) after the evaporation-induced self-assembly of CNCs, carbon dots and poly(vinyl alcohol) (PVA)^258^. CNCs/PVA could prevent the non-radiative relaxation by forming hydrogen bonds with carbon dots and stabilize the triplet state excitons, whereas carbon dots can foster the intersystem crossing (ISC) to populate triplet excitons that were protected and relaxed within the chiral matrix. It was found that CPL appeared under UV excitation while CPRTP occurred after removal of the excitation light, and therefore, both CPL and CPRTP could be facilely modulated by the photonic bandgaps of the hybrid nanostructured films. For example, right-handed CPL was induced when the emission of carbon dots was located at the center of the photonic bandgap, and left-handed CPL was induced when the emission of carbon dots was far away from the photonic bandgap. To prevent the phase separation and aggregation-caused quenching (ACQ) of carbon quantum dots in the CNC template, Xiong et al. reported the design and synthesis of nanocellulose/quantum dot nanostructured building blocks, and demonstrated flexible chiral luminescent nanomaterials with g_lum_ of -0.2 and robust mechanical properties^259^. Similarly, Chekini et al. reported chiral carbon dots synthesized on CNCs using a hydrothermal method and obtained chiral luminescent suspensions with left-handed CPL toward their potential application as biocompatible nanolabels for bioimaging^260^.

### Chiral perovskite nanocrystals

**Fig. 9:**
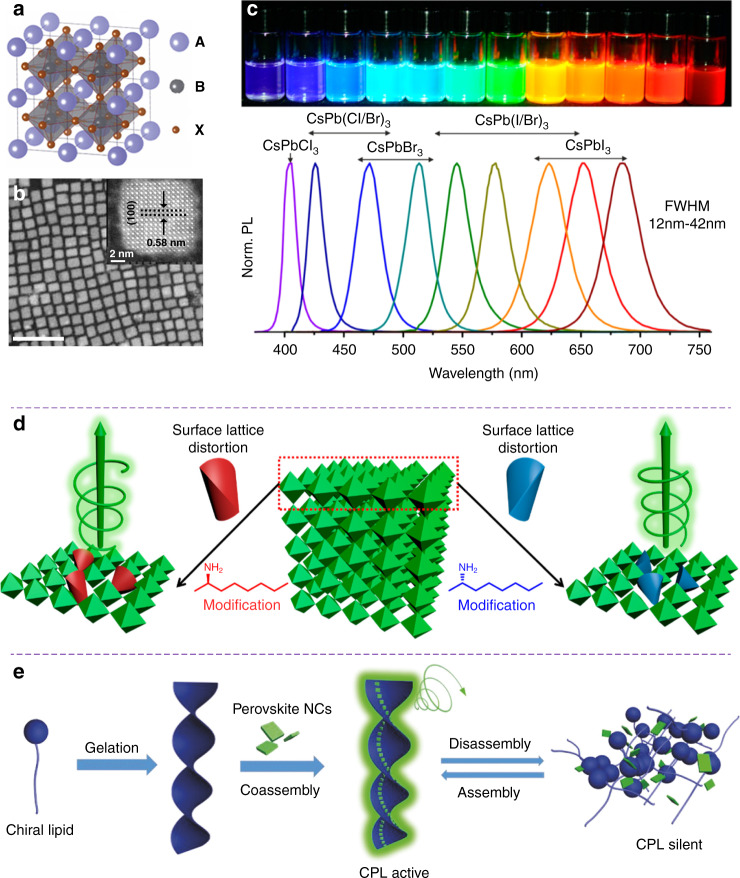
Perovskite nanocrystals and circularly polarized luminescence. **a** Schematic structure of perovskite nanocrystals. Reproduced with permission from ref. ^264^. Copyright 2015, American Chemical Society. **b** Typical TEM image of perovskite nanocrystals (Scale bar, 50 nm). Reproduced with permission from ref. ^262^. Copyright 2020, Springer Nature. **c** Colloidal perovskite nanocrystals exhibiting tailorable photonic bandgap across the entire visible spectral region. Reproduced with permission from ref. ^264^. Copyright 2015, American Chemical Society. **d** Chiral perovskite nanocrystals based on the chiral-ligand-assisted tip-sonication method. Reproduced with permission from ref. ^268^. Copyright 2019, American Chemical Society. **e** Chiral perovskite nanocrystals based on supramolecular soft templates. Reproduced with permission from ref. ^35^. Copyright 2018, WILEY-VCH

Perovskites have become promising optoelectronic materials suitable for different applications such as solar cells, photonic lasers, light­-emitting diodes (LEDs), and photodetectors^261^. Perovskites feature the general formula ABX_3_, where A is a monovalent cation of methylammonium (MA), formamidinium (FA), or Cs; B is a metallic cation of Pb or Sn; X is a halogen either Cl, Br, or I (Fig. 9a, b)^262^. Perovskite nanocrystals (PNCs) are known to exhibit many exceptional properties, including tailorable crystal nanostructures, high charge carrier mobilities, long electron-hole diffusion length, highly tunable bandgaps, strong spin-orbit couplings, large optical­ absorption coefficients, and low exciton binding energies^263^. The optical absorption and emission spectra of colloidal perovskite nanocrystals can be easily tailored over the entire visible spectral region by modulating their composition and nanoparticle size, which is also known as quantum-size effects (Fig. 9c)^264^. The first report of chirality in perovskites was 1D chiral perovskite nanocrystals in 2003^265^, and 2D chiral perovskite nanocrystals in 2006^266^. However, it was not until 2017 that the chiroptical properties of chiral perovskites were investigated in details^267^, and since then chiral perovskites have attracted enormous research attention. In 2019, Chen et al. reported that the chirality of perovskite nanocrystals could originate from the surface distortion induced by the chiral ligands (Fig. 9d)^268^. Interestingly, supramolecular self-assembly strategy has also been employed to construct chiral perovskite nanocrystals. For example, Shi et al. demonstrated that the chirality could be generated from surface-modified perovskite nanocrystals via gelator molecules (Fig. 9e)^35^. Chiral gelators and colorful perovskite nanocrystals could autonomously self-organize into chiral nanotubes in nonpolar solvents, displaying strong mirror-image CPL signals in the full-color range. It is worth noting that strong CD and CPL emission signals with a *g*_lum_ exceeding 6 × 10^−3^ have been achieved in chiral nanostructured films with helical arrangement of perovskite nanocrystals along the silica helical ribbons^269^.

**Fig. 10:**
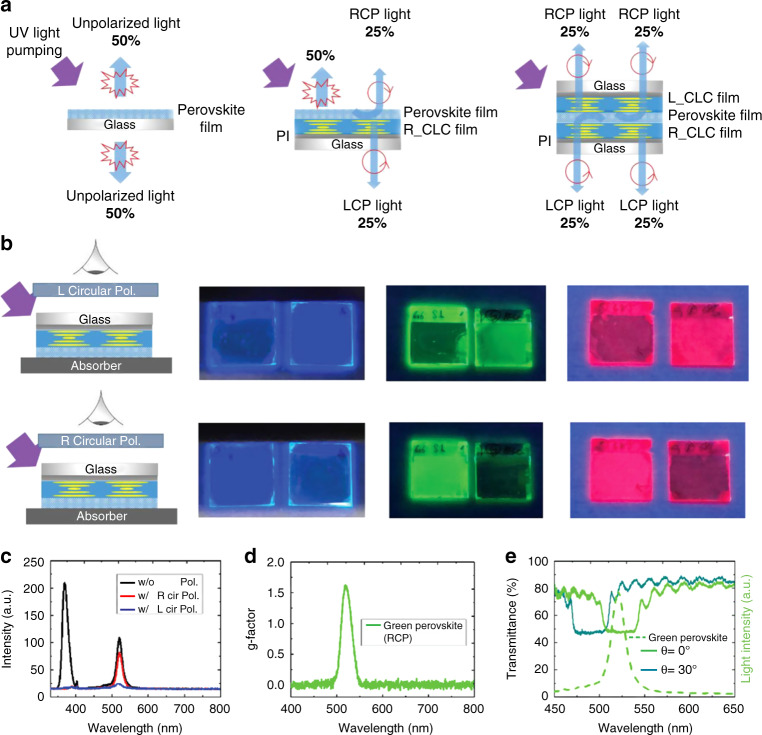
Chiral perovskite nanocrystals based on chiral liquid-crystalline template. **a** The principle of CPL induced by the cholesteric superstructure. **b** Photographs of liquid crystal-templated chiral perovskite films observed through a right-handed circular polarizer and a left-handed circular polarizer, respectively. **c** Luminescent spectra for chiral perovskite film exhibiting green luminescence probed with and without the polarizer. **d** *g*-factor for right-handed CPL from green chiral perovskite film. **e** The emission spectrum of a green chiral perovskite film and the reflection spectra of chiral liquid-crystalline film observed at 0° and 30° viewing angle. Reproduced with permission from ref. ^270^. Copyright 2019, WILEY-VCH

Recently, Li et al. reported the judicious design and synthesis of liquid crystal-templated chiral perovskite nanocrystals through the placement of perovskite layer between two chiral liquid-crystalline layers with predefined opposite handedness, and the resulting chiral perovskite nanosystems showed intense CPL emission and significantly enhanced *g*_lum_ (Fig. 10a)^270^. Thanks to the close relation between the CPL emission and the CLC nanostructured layers, the photonic bandgap of the chiral liquid-crystalline templates could be tailored to overlap with the emission of perovskites for achieving a high-*g*_lum_ value. Taking the right-handed CPL as an example, half of the right-handed CPL directly passed through the left-handed chiral liquid-crystalline layer, while the rest was reflected by the right-handed chiral liquid-crystalline lower layer and subsequently passed through the left-handed chiral liquid-crystalline layer. When a right-handed circular polarizer was placed between the left-handed chiral liquid-crystalline side and the detector, the measured right-handed CPL showed the same peak wavelength but lower intensity compared to the original emission. However, no emission and close-to-zero intensity were obtained when a left-handed circular polarizer was applied. As a result, an achiral perovskite film could be endowed with intense CPL emission with a high-*g*_lum_ value of 1.6. It was also found that the central wavelength of the reflection band exhibited a blue-shifting as the angle of incidence increased, which reduced the overlap of the reflection band and the light emission, and thus, the CPL intensity was highly dependent on the viewing angle (Fig. 10b–e). The chiral perovskite nanomaterials with tailorable and tunable CPL properties could find many emerging optoelectronic applications such as spintronics, 3D displays, quantum computation and beyond. Despite great potential of perovskite nanocrystals in CPL-active nanomaterials and optoelectronic devices, their poor stability remains a daunting problem that hampers their practical application. Perovskite nanocrystals with the dynamic surface are highly sensitive to the environment, such as polar solvents, light, oxygen and heat, due to their intrinsically ionic nature, high surface energy, and easy migration of surface ligands^271^. Many promising strategies have been developed to improve the stability of perovskite nanocrystals, including surface chemistry engineering, compositional engineering, matrix encapsulation and device encapsulation^272,273^. For example, Liu et al. found that polyacrylonitrile (PAN) encapsulation could substantially improve luminescent efficiency and long-term stability of perovskite in a polar environment. Stable CPL with a *g*_lum_ value up to 1.9 was further demonstrated by lamination of chiral liquid-crystalline templates. The resulting CPL-active bilayer devices with various graphical patterns and reversible thermal-switching behavior show possibilities for cryptology and anti-counterfeiting applications^274^. It should be noted that liquid crystal-templated long-range ordered assembly of perovskite nanocrystals has not been reported yet, and the realization of stable dispersion and ordered assembly of perovskite nanocrystals in chiral liquid-crystalline templates could open many new opportunities in chiral luminescent nanomaterials.

### Chiral upconversion nanoparticles

**Fig. 11:**
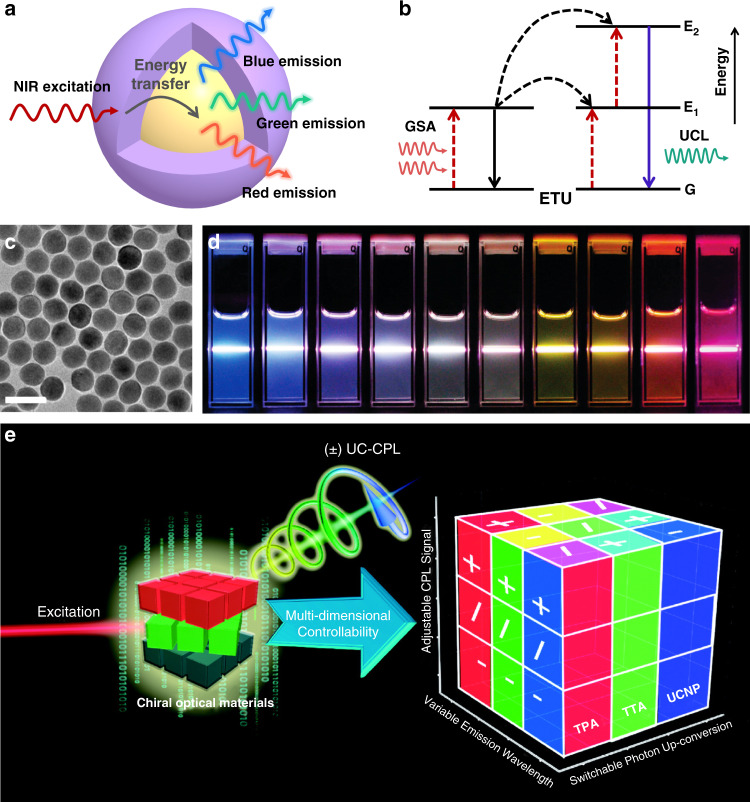
Upconversion nanoparticles and their optical properties. **a** Schematic depiction of UCNPs. **b** ETU process of activators and sensitizers in UCNPs. **c** Typical TEM image of UCNPs (Scale bar, 50 nm). Reproduced with permission from ref. ^291^. Copyright 2019, Springer Nature. **d** Tunable emissions of UCNPs excited with NIR light. Reproduced with permission from ref. ^292^. Copyright 2008, American Chemical Society. **e** Schematic representation for multi-dimension information involved in chiral upconversion nanomaterials, including the CPL signal, the wavelength of emission, and excitation in photon upconversion. Reproduced with permission from ref. ^293^. Copyright 2021, The Chemical Society of Japan

Upconversion nanoparticles (UCNPs) are known to be capable of converting low-energy-absorbed photons into high-energy photon emissions via long-lived intermediate energy states of lanthanide ions^275–277^. Unlike conventional luminescent quantum dots, UCNPs have many outstanding advantages, such as large anti-Stokes shifts, negligible auto-fluorescence background, sharp emission peaks, low toxicity and high resistance to photo-bleaching. It should be noted that UCNPs show low light scattering and allow deep penetration of tissues because their excitation occurs in the near-infrared (NIR) region, which is within the optical transparency window^278^. All of these merits render UCNPs particularly useful in diverse emerging applications, such as photoswitching, biosensing, bioimaging, and advanced information displays^279–281^. For UCNPs, their low luminescence brightness, low extinction coefficients as well as fixed energy levels remain major challenges for unlocking their full potential^282^. Over the past decade, considerable efforts have been made in the judicious design and synthesis of lanthanide-based UCNPs exhibiting robust photoluminescence properties^283^. Lanthanide-based UCNPs are typically made of a crystalline host and a lanthanide-ion dopant added in a low concentration (Fig. 11a), in which the dopant acts as luminescent centers while the host lattice offers a crystalline matrix to bring these centers into optimal positions^284^. UCNPs usually absorb two or more low-energy photons upon NIR irradiation and emit high-energy photons in the UV or visible range via nonlinear anti-Stocks optical process, i.e., upconversion luminescence^285^. Excited-state absorption (ESA) and energy-transfer upconversion (ETU) are the most efficient mechanisms by which the upconversion process is carried out in lanthanide-based UNCPs^286^. In the case of ESA, the emitting ions in excited state sequentially absorb at least two additional photons of suitable energy to reach higher excited state^287,288^. As for ETU, one photon is absorbed by the ion, but subsequent energy transfer between neighboring ions results in the population of a highly excited state of the emitting ion (Fig. 11b)^289,290^. Most of UCNPs have spherical and dumbbell shapes with core diameters ranging from around 5 to 50 nm (Fig. 11c)^291^. Moreover, by changing the categories of doped lanthanide ions and the particle size, their luminescence emission can be facilely tuned from the visible to the NIR range under single-wavelength excitation (Fig. 11d)^292^. Considering the polarization of light, upconverted circularly polarized luminescence can be obtained by modulating the polarization state of upconverted luminescence (Fig. 11e)^293^. In general, CPL displays the chiroptical properties of chiral luminescent system at the excited state; therefore, higher-energy excitation is needed in most of the developed chiral nanomaterials. Consequently, this type of down-converted CPL has some shortcomings, such as only photons with an energy higher than the HOMO-LUMO gap energy of the emitters can be utilized^294,295^. Unlike the down-conversion process, the coupling of CPL and photon upconversion enables the conversion of unpolarized lower-energy photons into circularly polarized higher-energy photons through a sequence of energy-transfer steps^296,297^. Extensive research has been carried out to achieve upconverted CPL, and they can be divided into three categories: two-photon absorption upconversion (TPA-UC)^268^, triplet-triplet annihilation-based upconversion (TTA-UC)^296,298–300^, as well as lanthanide-doped upconversion nanoparticles with hybrid mechanisms^301,302^. For example, Jin et al. encapsulated two kinds of achiral UCNPs into chiral nanotubes assembled from chiral gelator as host, and the upconverted CPL with *g*_lum_ around 5.48 × 10^−3^ was achieved^36^.

**Fig. 12:**
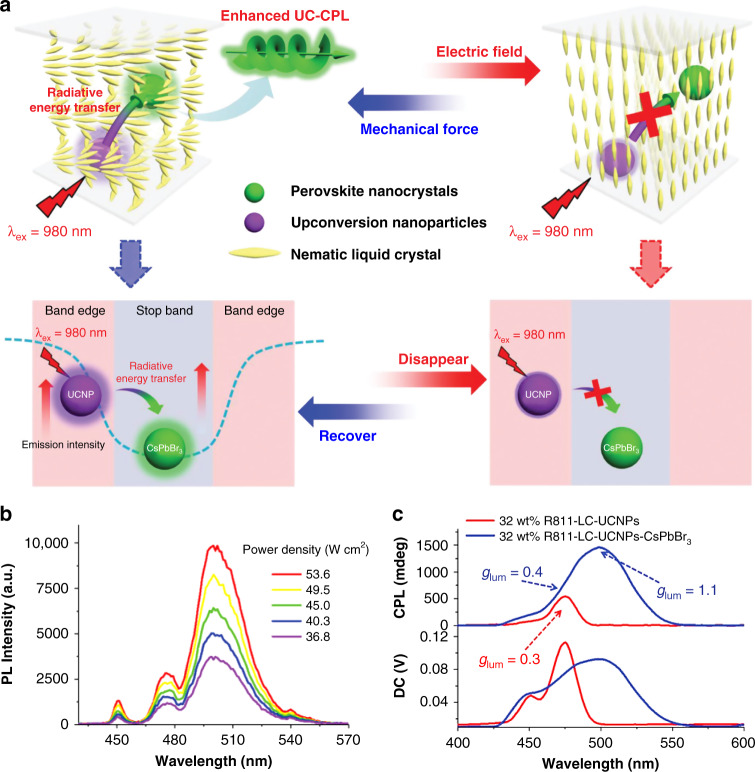
Chiral upconversion nanoparticles based on chiral liquid-crystalline template. **a** Schematic illustration of switchable CPL in chiral UCNPs under electric field. **b** Upconversion emission spectra of chiral UCNPs with different incident excitation intensities of 980 nm NIR light. **c** Upconversion CPL spectra of chiral UNCPs upon NIR irradiations. Reproduced with permission from ref. ^304^. Copyright 2020, WILEY-VCH

Recently, chiral liquid-crystalline templates have been used to amplify the *g*_lum_ value of the chiral upconversion nanomaterials. Li et al. reported chiral upconversion nanomaterials based on CNC liquid-crystalline template, in which a tunable upconverted CPL emission was obtained with a relatively high tailored *g*_lum_ ranging from −0.156 to −0.033^303^. Thanks to the strong hygroscopicity of CNC template, the photonic bandgap of the chiral luminescent nanomaterials exhibited an evident red-shifting upon increasing the relative humidity. Decreasing the overlap of the upconverted emission with the photonic bandgap led to an obvious decrease in the upconverted CPL. As a result, the resulting chiral luminescent film exhibited an upconverted CPL with different *g*_lum_ values under different humidity conditions. Yang et al. demonstrated a new strategy to achieve enhanced upconverted CPL via the energy-transfer process from UCNPs to the perovskite nanocrystals in chiral liquid-crystalline template (Fig. 12a–c)^304^. The perovskite nanocrystals, which served as energy acceptors, allowed to obtain a higher *g*_lum_ value by taking advantage of the intense reflection found at the center of the photonic bandgap of chiral liquid-crystalline template. It was found that enhanced emission of the UCNPs was located at the edge of the photonic bandgap, which could be further reabsorbed by perovskite nanocrystals, thus resulting in improved emission of perovskite nanocrystals. An upconverted CPL with high-*g*_lum_ up to 1.1 was achieved in the resulting chiral luminescent nanomaterials through appropriately modulating the appropriate ratio of donor and acceptor for ensuring the highest efficiency of radiative energy transfer. It is worth noting that the emission of upconverted CPL and the process of radiative energy transfer could be facilely switched off upon applying an electric field, while it would recover to the original state upon applying mechanical force. Moreover, Guo et al. demonstrated NIR light-driven reversible modulation of CPL accompanied by a change in *g*_lum_ through introducing UCNPs and a photoswitch in the chiral liquid-crystalline template^305–307^.

**Fig. 13:**
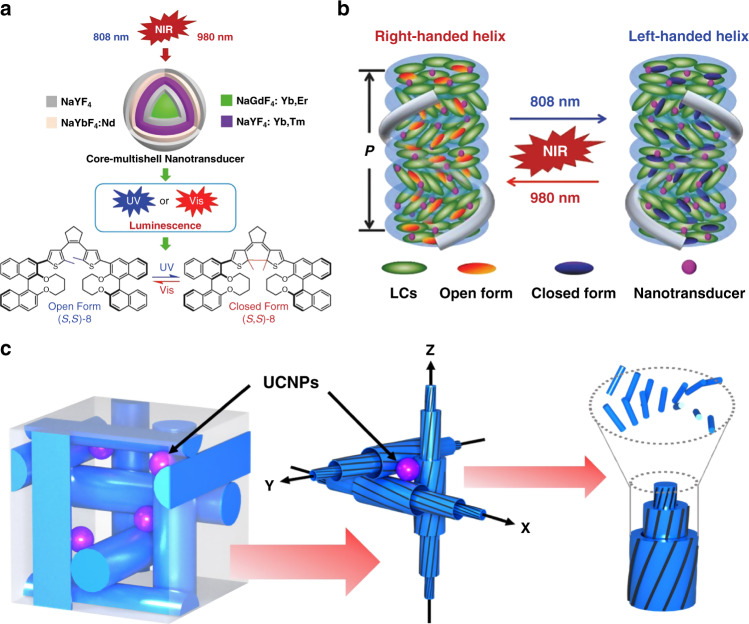
NIR light-driven chiral liquid crystal nanostructures with UCNPs. **a** Schematic of NIR light-driven reversible switching of a unique chiral diarylethene with core-multishelled UCNPs. **b** Schematic demonstration of NIR light-driven handedness inversion of cholesteric superstructures enabled by UCNPs and a chiral diarylethene photoswitch. Reproduced with permission from ref. ^309^. Copyright 2015, WILEY-VCH. **c** Blue-phase 3D nanostructure stabilized by UCNPs. Reproduced with permission from ref. ^310^. Copyright 2022, WILEY-VCH

It should be noted that UCNPs can be used as emerging NIR-to-UV/Vis nanotransducers for controlling the self-organization of superstructures of chiral liquid crystals loaded with light-driven molecular photoswitches^308–310^. For instance, Wang et al. demonstrated NIR photoresponsive chiral liquid-crystalline superstructures loaded with a novel chiral azobenzene-based molecular photoswitch and unique NaGdF_4_:Yb,Tm@NaGdF_4_ UCNPs. NIR light-driven dynamic and reversible modulation of photonic bandgap across the entire visible spectrum was achieved by changing the 980 nm NIR light intensity^308^. Interestingly, NIR photoswitchable self-organized helical superstructures were also demonstrated by doping core-multishell UCNPs with a novel chiral dithienylethene-based molecular photoswitch. Unprecedented reversible handedness inversion of chiral liquid-crystalline nanostructures was then achieved by selective and alternating exposure to NIR light with different wavelengths of 980 and 808 nm (Fig. 13a, b)^309^. Recently, Qiu et al. reported NIR light-driven dynamic and reversible photonic bandgap modulation of blue-phase liquid crystals with chiral 3D nanostructures through the integration of a chiral azobenzene photoswitch and novel core-shell UCNPs (Fig. 13c)^310^. A red-shift of the photonic reflection towards a longer wavelength was observed upon irradiating the chiral 3D nanostructures with a 808 nm light at high-power density, while blue-shifting took place upon 808 nm NIR light irradiation at lower power density. This research can provide insights into the large-scale fabrication of programmable and reconfigurable chiral 3D photonic nanostructures towards different applications such as soft-matter photonics and future integrated communication technologies.

To summarize the above discussion, an overview of chiral liquid-crystalline templates, fluorescent nanomaterials, *g*_lum_, and applications of chiral luminescent nanomaterials is presented in Table 1. It is very clear that liquid crystal-templated chiral luminescent nanomaterials exhibit a significantly enhanced CPL with *g*_lum_ > 10^−1^. However, the enhancement of *g*_lum_ value varies for different chiral luminescent nanomaterials. For example, the maximum *g*_lum_ of lyotropic CNC-templated chiral carbon dots is −0.74, while the *g*_lum_ of lyotropic CNC-templated UCNPs is only −0.156. The *g*_lum_ of chiral luminescent nanomaterials could be affected by diverse factors such as the compatibility of liquid-crystalline templates with fluorescent nanomaterials, the concentration of fluorescent nanomaterials, and the orientational alignment of liquid-crystalline templates. Moreover, the differences in *g*_lum_ values could also result from the non-uniform operating conditions and methods, and the establishment of uniform test standards for evaluating *g*_lum_ values should be of paramount significance for the future development of advanced chiral luminescent nanomaterials. It should be also noted that the ACQ is often unavoidable when embedding high concentration of inorganic quantum dots into chiral liquid-crystalline templates, which results in poor chiroptical properties and low-*g*_lum_. In this context, many strategies have been proposed to overcome this limitation. The first strategy is to apply the fluorescent nanomaterials as a light-emitting layer to avoid the low compatibility of inorganic quantum dots in chiral liquid-crystalline templates, and a high-*g*_lum_ value of ~1.6 has been demonstrated by such a stacking strategy^270^. The second strategy is to design and synthesize mesogenic ligand-functionalized quantum dots to improve their miscibility in chiral liquid-crystalline templates. The most promising strategy may be to develop quantum dots exhibiting aggregate-induced emission (AIE) effect to overcome the ACQ^311,312^. Recently, many efforts have been devoted to introduce organic luminogens with AIE properties into chiral liquid-crystalline templates^171,313–317^. By supramolecular self-assembly between achiral AIE guests and chiral liquid-crystalline templates, these compounds exhibit strong CPL signals and a large *g*_lum_ value of 1.42 has been reported^318^.

**Table 1:** Chiral luminescent nanomaterials based on chiral liquid-crystalline templates

Chiral liquid-crystalline template	Fluorescent nanomaterials	|*g*_lum_|	Application	Ref.
Lyotropic CNC	ZnS/CdSe QDs	0.48	Optical labels	^68^
Lyotropic CNC	CdSe/CdS QRs	0.45	Semiconductor quantum materials with CPL	^247^
Lyotropic CNC	CdSe/CdS QNRs	0.2	Mechanically triggered dynamic light polarization	^250^
Lyotropic CNC	Carbon QDs	0.2	Chiral fluorescent patterns	^259^
Lyotropic CNC	Carbon dots	0.27	Circular polarized room-temperature phosphorescence	^258^
Lyotropic CNC	Carbon dots	0.74	Circularly polarized light detection	^257^
Lyotropic CNC	Carbon dots	0.2	Biotags	^260^
Lyotropic CNC	NaYF_4_:TmYb UCNPs	0.156	Humidity-responsive CPL	^303^
Thermotropic CLCs	CdSe QDs	~0.8	Optically/electrically controlled CPL	^246^
Thermotropic CLCs	CdSe/ZnS QDs	~1.5	Optically/electrically controlled CPL	^244^
Thermotropic CLCs	CdSe/ZnS QDs	~2	Phototunable CPL	^245^
Thermotropic CLCs	CsPbX_3_ PNCs	1.6	Optoelectronic devices	^270^
Thermotropic CLCs	MAPbBr_3_ PNCs	1.9	Cryptology	^274^
Thermotropic CLCs	UCNPs and CsPbBr_3_ PNCs	1.1	Electric-field controlled UC-CPL	^304^

## Conclusions and perspectives

Chiral soft templates provide a powerful and straightforward bottom-up self-assembly strategy for the design and synthesis of chiral nanomaterials with hierarchical architectures and advanced functionalities. Compared to emerging DNA-based soft templates, chiral liquid-crystalline templates are faster, less expensive, and more adaptable to guide the self-assembly of nanoscale building blocks into arbitrary and high-order chiral nanomaterials over a larger range of scales, thanks to their inherent long-range ordered molecular arrangements that combine the liquid fluidity with crystal ordering from atomic-molecular to macroscopic levels. In this review, we offer an account of the state-of-the-art advances on liquid crystal-templated chiral functional nanomaterials, including chiral plasmonic nanomaterials and chiral luminescent nanomaterials. Different thermotropic and lyotropic liquid crystal templates have been applied for fabricating chiral plasmonic nanomaterials with enhanced CD, amplified dissymmetry factor, and dynamic chiroptical responses, which are of paramount significance for many potential applications, such as negative-refractive-index materials, ultrasensitive biosensing, enantioselective analysis, advanced light-polarization filters, and beyond. A variety of emerging nanoscale functional building blocks, such as inorganic quantum dots, perovskite nanocrystals, and upconversion nanoparticles, have been employed for the design and synthesis of novel chiral luminescent nanomaterials exhibiting significantly enhanced circularly polarized luminescence, which could find important applications in many emerging fields, such as biological science, 3D display, information encryption, chiral spintronics, and enantioselective photochemistry.

Despite the great achievements, the development of chiral functional nanomaterials based on the bottom-up soft template strategy remains in its early stages, and there are still many challenges to be addressed for motivating breakthrough research in this significant field. The first one is to develop chiral functional nanomaterials with high optical asymmetry *g*-factors (*g*_abs_ for absorption and *g*_lum_ for luminescence), as the reported values are still far from the theoretical value of ±2. The second one is to endow the chiral functional nanomaterials with tunable chiroptical activity to diverse target wavelengths ranging from the ultraviolet, visible, near-infrared to terahertz regions. It is also very important to achieve real-time reconfigurable chiral functional nanomaterials that show ultrasensitive responsiveness to the external environment through the dynamic nature of chiral liquid-crystalline templates. For example, electroresponsive chiral liquid crystals with sub-microsecond response time are particularly interesting for developing ultra-fast chiroptical devices^319^, and the marriage of chiral liquid-crystalline templates with advanced photoalignment technique and emerging chiral photoswitches could facilitate the breakthrough development of reconfigurable chiral functional nanomaterials^320–323^. Thirdly, extensive efforts should be devoted to bridging the research gap from the proof-of-concept on a laboratory scale to the large-scale synthesis of chiral functional nanomaterials and their integration into multi-material hierarchical architectures and even more complex advanced functional devices. Moreover, many other liquid-crystalline phases^324,325^, such as 2D chiral smectics and 3D blue phases, and inorganic chiral photonic crystals^326^, can be applied for the development of chiral hierarchical nanomaterials with unprecedented functionalities. Chiral metal-organic frameworks (MOFs), as an emerging class of nanoporous chiral assemblies, could be one of the preeminent chiral template candidates due to their versatile performance in combination with diverse functional nanomaterials^327^. Owing to the arbitrary design capability, chiral functional nanomaterials can also be constructed with chiroptical properties that may not be readily revealed within their molecular counterparts, such as chirality-dependent mechanical properties^328^ and magnetically induced chiroptical properties^329^. Chiral functional nanomaterials that possess 3D stereochirality may act as a powerful platform for fundamental research of the chirality transfer and amplification between nanoscale building blocks and advanced bulk materials. The development of new characterization techniques can deepen our understanding of the enantiospecific and chiroptical effects at the nano- or atomic scale^330^. The development of chiral spintronics based on chiral magnetic nanomaterials could pave an avenue for their important applications in areas of memory, logic, and memristic analog devices^331,332^. It is anticipated that the unique combination of liquid-crystalline nanoscience with nanoscale chirality and emerging bottom-up self-assembly will pour vitality into the development of programmable and reconfigurable chiral functional nanomaterials with unlimited possibilities. Future endeavors of scientists and engineers from multidisciplinary research backgrounds will certainly bring new twists into fundamental breakthrough and technological applications of emerging soft-matter chirality and truly advanced chiral functional nanomaterials which embrace biology, optics, electronics, spintronics, physics, chemistry, materials science, device engineering, and other interdisciplinary areas.
